# Antioxidant, Antimicrobial and Cytotoxic Properties as Well as the Phenolic Content of the Extract from *Hancornia speciosa* Gomes

**DOI:** 10.1371/journal.pone.0167531

**Published:** 2016-12-01

**Authors:** Uilson P. Santos, Jaqueline F. Campos, Heron Fernandes V. Torquato, Edgar Julian Paredes-Gamero, Carlos Alexandre Carollo, Leticia M. Estevinho, Kely de Picoli Souza, Edson Lucas dos Santos

**Affiliations:** 1 School of Environmental and Biological Science, Federal University of Grande Dourados, Rodovia Dourados Ithaum, Dourados, MS, Brazil; 2 Department of Biochemistry, Federal University of São Paulo, São Paulo, SP, Brazil; 3 Center for Interdisciplinary Research Biochemistry, University of Mogi das Cruzes, Av. Dr. Cândido Xavier de Almeida Souza, Mogi das Cruzes, SP, Brazil; 4 Federal University of Mato Grosso do Sul, Center of Biological and Health Sciences, Laboratory of Natural Products and Mass Spectrometry, Campo Grande, MS, Brazil; 5 Agricultural College of Bragança, Polytechnic Institute of Bragança, Campus Santa Apolónia, Bragança, Portugal and Centre of Molecular and Environmental Biology, University of Minho, Campus de Gualtar, Braga, Portugal; Institute of medical research and medicinal plant studies, CAMEROON

## Abstract

*Hancornia speciosa* Gomes (Apocynaceae) is a fruit tree, popularly known as mangabeira, and it is widely distributed throughout Brazil. Several parts of the plant are used in folk medicine, and the leaf and bark extracts have anti-inflammatory, antihypertensive, antidiabetic, and antimicrobial properties. In this study, we investigated the chemical composition of the ethanolic extract of *Hancornia speciosa* leaves (EEHS) and its antioxidant, antimicrobial, and cytotoxic activities as well as the mechanisms involved in cell death. The chemical compounds were identified by liquid chromatography coupled to mass spectrometry (LC-MS/MS). The antioxidant activity of the EEHS was investigated using the method that involves the scavenging of 2,2-diphenyl-1-picrylhydrazyl free radicals as well as the inhibition of oxidative hemolysis and lipid peroxidation induced by 2,2’-azobis (2-amidinopropane) in human erythrocytes. The antimicrobial activity was determined by calculating the minimum inhibitory concentration, minimum bactericidal concentration, minimum fungicidal concentration, and zone of inhibition. Kasumi-1 leukemic cells were used to assess the cytotoxic activity and mechanisms involved in cell death promoted by the EEHS. The chemical compounds identified were quinic acid, chlorogenic acid, catechin, rutin, isoquercitrin, kaempferol-rutinoside, and catechin-pentoside. The EEHS demonstrated antioxidant activity via the sequestration of free radicals, inhibition of hemolysis, and inhibition of lipid peroxidation in human erythrocytes incubated with an oxidizing agent. The antimicrobial activity was observed against American Type Culture Collection (ATCC) and hospital strains of bacteria and fungi, filamentous fungi and dermatophytes. The cytotoxic activity of the EEHS was induced by apoptosis, reduction of the mitochondrial membrane potential, and activation of cathepsins. Together, these results indicate the presence of phenolic compounds and flavonoids in the EEHS and that their antioxidant, antimicrobial, and cytotoxic activities in acute myeloid leukemia cells are mediated by apoptosis.

## Introduction

The cerrado region (Brazilian Savannah) of Brazil covers approximately 2 million km^2^ and corresponds to approximately 22% of the Brazilian territory [1]. This biome has a wide variety of medicinal plants used in folk medicine. Many of these plants have been investigated, and their antioxidant [2], antimicrobial [3], antidiabetic [4], anti-inflammatory [5], and cytotoxic [6] activities, among others, have been scientifically proven.

One of the medicinal plants found in the Brazilian cerrado is *Hancornia speciosa* Gomes (Apocynaceae), popularly known as mangabeira. In this species, the roots have antihypertensive and wound-healing activities [7]; the bark has antidiabetic, anti-obesity, antimicrobial, and gastroprotective activities [3,8,9]; the latex has anti-inflammatory activity [10]; and the leaves have antihypertensive [11], vasodilator [12,13], anti-inflammatory [14,15], and antidiabetic [4] activities and are used for treatment of dysmenorrhea [16].

Presently, there is a growing demand for natural products with therapeutic activities, including antioxidant activity, which can overcome the harmful effects of free radicals [17], and low toxicities compared with synthetic antioxidants that are widely used in food products, cosmetics, and drugs [18,19].

Among the main chemical compounds responsible for the antioxidant activities of medicinal plants, phenolic compounds and flavonoids are the most prominent because of their roles against oxidative stress [20,21].

These compounds also have antimicrobial activities [22]. These properties have attracted scientific interest because 60% of the antimicrobial drugs discovered in the past few decades are of natural origin [23]. Furthermore, the number of pathogens that are resistant to commercial antimicrobials has increased [24].

In addition, compounds derived from natural sources have great potential as anticancer drugs, and 51% of the drugs currently available for treatment of this pathology are directly or indirectly derived from natural products [23]. Among these compounds, phenolic compounds and flavonoids from several plant species have cytotoxic activities against different cell lines, including leukemic cell lines [2,25,26].

In this context, the aim of this study was to determine the chemical composition of the ethanolic extract of *Hancornia speciosa* Gomes leaves and evaluate its antioxidant, antimicrobial, and cytotoxic activities *in vitro* using the acute myeloid leukemia cell line Kasumi-1.

## Materials and Methods

### Ethics of Experimentation

The *H*. *speciosa* Gomes leaves were collected following the identification of the plant and authorization of the SISBIO *(Sistema de Autorização e Informação em Biodiversidade*, permit number 54470–1). The protocol to collect of human peripheral blood, was approved by the Research Ethics Committee (Comitê de Ética em Pesquisa; CEP) of the University Center of Grande Dourados, Brazil (CEP process number 123/12). All subjects provided written informed consent for participation.

### Plant Material and Extract Preparation

The *H*. *speciosa* Gomes leaves were in Dourados, Mato Grosso do Sul (S 21°59’ 41” and W 55°19’ 24”), Brazil, oven-dried with the air circulation at a temperature of 45 ± 5°C, and then ground in a Willy-type knife mill. An exsiccated sample was deposited in the Herbarium of the Federal University of Grande Dourados, Mato Grosso do Sul, Brazil, with registration number 4774.

The extract was then prepared by macerating the plant material in an ethanol 96% (1:10) mixture at room temperature for 14 days. Then, the extract was filtered, the filtrate was concentrated in a rotary vacuum evaporator (Gehaka, São Paulo, SP, Brazil), freeze-dried to obtain a calculated specific yield of 28%, and the final freeze-dried ethanol extract of *H*. *speciosa* Gomes (EEHS) was stored at—20°C protected from light.

### Chemical Analysis

#### Determination of total flavonoids and phenolic compounds

The content of phenolic compounds in the EEHS was determined using the Folin-Ciocalteu colorimetric method, as detailed by Meda et al. [27], with some modifications. The EEHS (200 μg/mL) was diluted in absolute ethanol, and a 0.5-mL aliquot was added to 2.5 mL of Folin-Ciocalteu reagent (diluted 1:10 with distilled water). This solution was allowed to stand for 5 min at room temperature. After this period, 2 mL of a 14% sodium carbonate solution was added to the samples, the mixture was incubated for 2 h at room temperature, and the absorbance was read at 760 nm in a T 70 UV/VIS spectrophotometer (PG Instruments Limited, UK). A standard curve was prepared using gallic acid in the concentration range of 0.4–11.0 μg/mL. The total amount of phenolic compounds was expressed in milligrams of gallic acid equivalents (GAE) per gram of the EEHS.

The total flavonoids present in the EEHS were determined according to the method described by Liberio et al. [28], with some modifications. Briefly, 4.5 mL of a hexahydrate aluminum chloride solution (AlCl_3_·6H_2_O) at 2% in absolute methanol was mixed with 0.5 mL of the EEHS (200 μg/mL). The mixture was incubated for 30 min at room temperature, and the absorbance was read at 415 nm in a T 70 UV/VIS spectrophotometer (PG Instruments Limited, UK). A standard curve was prepared using quercetin in the concentration range of 0.4–11.0 μg/mL. The total flavonoids were expressed in milligrams of quercetin equivalents (QE) per gram of the EEHS. All experiments were performed in triplicate.

#### Determination of phenolic compounds in the EEHS using HPLC-DAD-MS/MS

A 1μL aliquot of the EEHS (1 mg/mL) was analyzed via high-performance liquid chromatography with diode-array detection (HPLC-DAD; Shimadzu, Japan) coupled to a high-resolution mass spectrometer (model micrOTOF-Q IIII, Bruker, Germany). A C-18 column (Kinetex, 2.6 μ, 150 x 2.2 mm) protected by a pre-column of the same material was used. The mobile phases used were water (phase A) and acetonitrile (phase B), both containing 1% acetic acid. The following gradient elution was used: 0–2 min. at 5% B, 2–20 min. at 5%–80% B, and an additional 10 min for column washing and re-equilibration. The flow rate was 0.2 mL/min. The mass spectrometer parameters were as follows: capillary voltages of the electrospray ionization system (ESI) of 3500 V (negative mode) and 4500 V (positive mode). The capillary temperature was 200°C, the collision energy was variable for the MS/MS experiments, and data were obtained in the positive and negative ion modes. The negative mode was chosen because it generated more data than the positive mode. Trifluoroacetic acid sodium salt was used as the internal calibrant. The compounds quinic acid, chlorogenic acid, catechin, rutin, and isoquercitrin were identified by comparison with commercial standards (Sigma-Aldrich).

### Antioxidant activity

#### Scavenging of DPPH free radicals

The scavenging of stable DPPH radicals was evaluated using the method detailed by Gupta and Gupta [29], with some modifications. Briefly, 200 μL of the EEHS (100–1000 μg/mL) was mixed with 1.8 mL of an ethanol solution of 0.1 mM DPPH. The mixture was homogenized and incubated for 30 min at room temperature in the dark. The absorbance was read at 517 nm in a T 70 UV/VIS spectrophotometer (PG Instruments Limited, UK). Ascorbic acid and butylated hydroxytoluene (BHT) were used as the antioxidant standards. Three independent experiments were performed in duplicate. The percentage of free radical-scavenging activity was expressed with the following formula: 1—(Abs_sample_/Abs_control_) x 100.

### Antioxidant assay in human erythrocytes

#### Inhibition assay of AAPH-induced hemolysis

The protection against hemolysis promoted by the EEHS were evaluated using the method described by Valente et al. [30], with some modifications. The tests were conducted with erythrocyte suspensions (2.5%) previously incubated at 37°C for 30 min in the presence of different concentrations of ascorbic acid or the EEHS (50–125 μg/mL). After this period, 2.2’-azobis-2-amidinopropane (AAPH) at a concentration of 50 mM was added to the samples subjected to hemolysis induction. This mixture was incubated at 37°C for 4 h with frequent stirring. The negative control consisted of erythrocytes incubated with ethanol at a final concentration of 0.6%. After every 60-min incubation period, samples were centrifuged at 1500 rpm for 10 min, and an aliquot of each supernatant was collected and diluted in saline solution. Subsequently, the absorbance was read at 540 nm in a T 70 UV/VIS spectrophotometer (PG Instruments Limited, UK). Three independent experiments were conducted in duplicate. The percentage of hemolysis was measured using the following formula: A/B x 100, where (A) corresponds to the sample absorbance and (B) corresponds to the total hemolysis (erythrocytes incubated with distilled water).

#### Inhibition of malondialdehyde production

The effect of the EEHS on the inhibition of the production of malondialdehyde (MDA), a by-product of lipid peroxidation, was evaluated by incubation of a human erythrocyte suspension (5%) in 50 mM of the oxidizing agent AAPH [31]. The suspension was pre-incubated at 37°C for 30 min in the presence of ascorbic acid or the EEHS (100 μg/mL). Afterwards, 50 mM AAPH was added, and the mixture was maintained at 37°C for 3 h with frequent stirring. The negative control consisted of erythrocytes incubated with ethanol at a final concentration of 0.6%. After the incubation period, the samples were centrifuged at 1500 rpm for 10 min. Aliquots (500 μL) of the supernatants were transferred to test tubes containing 1 mL of 10 nmoL thiobarbituric acid (TBA). The standard solution consisted of 500 μL of 20 mM MDA in 1 mL of TBA. The samples were incubated at 96°C for 45 min. After the samples were cooled for 15 min, 4 mL of n-butyl alcohol was added to each sample, and the samples were centrifuged at 3000 rpm for 5 min. The supernatants were read at 532 nm in a T 70 UV/VIS spectrophotometer (PG Instruments Limited, UK). Two independent experiments were conducted in triplicate. The MDA levels in the samples were expressed in nmol/mL using the following formula: sample absorbance x (20 x 220.32/standard absorbance).

### Antimicrobial activity

#### Microbial growth

The antimicrobial activity of the EEHS was evaluated using gram-negative bacteria (*Klebsiella pneumoniae* and *Proteus mirabilis*), gram-positive bacteria (*Staphylococcus aureus*), and the yeast *Candida albicans*. All microorganisms used were certified by the American Type Culture Collection (ATCC) and the Agricultural School (*Escola Superior Agrária*, *ESA*) of Bragança, Portugal. The tested strains were initially cultured in Muller-Hinton broth containing 20% glycerol and were stored at –70°C. Before experimental use, each sample was subcultured overnight in liquid nutrient broth (for the bacterial strains) or peptone dextrose liquid medium (for the yeast strain). The inocula were diluted in saline solution and adjusted to a 0.5 McFarland standard. Each dilution was confirmed by spectrophotometric readings at 540 nm for bacterial strains and 640 nm for yeast strains in a Unicam Helios Alpha UV-VIS spectrophotometer (Thermo Spectronic, Cambridge, UK) [32]. Aliquots of different bacterial inocula (10^8^ colony forming units (CFU)/mL) and yeast inocula (10^5^ CFU/mL) were added to the microplates for evaluation of antimicrobial activity.

#### Minimum inhibitory concentration

The minimum inhibitory concentrations of the EEHS as well as the controls, gentamicin (antibiotic) and amphotericin B (antifungal), were determined using a microdilution assay, as described by Morais et al. [33]. Antimicrobial assays were conducted using nutrient broth (NB) (for the bacterial strains) or yeast peptone dextrose (YPD) liquid medium (for the yeast strain) in 96-well microplates. The EEHS was solubilized in dimethylsulfoxide (DMSO) at 70% (final concentration of DMSO of 0.27%), and serial dilutions were prepared with concentrations between 0.78 and 100 mg/mL. The inocula were added to all wells except for the negative controls, and the microplates were incubated at 37°C for 24 h (for the bacterial strains) or 25°C for 48 h (for the yeast strain). DMSO was used as the control. The antimicrobial activity was detected by adding 20 μL of 1% 2,3,5-triphenyl-2H-tetrazolium (TTC). The minimum inhibitory concentration (MIC) was defined as the lowest concentration of the sample capable of inhibiting microbial growth, as indicated by the TTC staining. The MIC was calculated after collecting a 20μL aliquot from each well where color changes were not observed and transferring it to NB or YPD medium for 24 h at 37°C (for the bacterial strains) or 48 h at 25°C (for the yeast strain). The lowest concentration that did not result in growth after subculturing was defined as the minimum bactericidal concentration (MBC) or minimum fungicidal concentration (MFC). Assays were performed in triplicate for each microorganism.

#### Inhibition of fungal growth

Pure cultures of fungal isolates were obtained from the Microbiology Laboratory of ESA. The agar diffusion technique was used to evaluate the activity of the EEHS against the filamentous fungi *Colletotrichum acutatum* ESA12, *Fusarium culmorum* ESA23, and *Mucor piriformis* ESA43, as well as the dermatophytes *Microsporum canis* ESA28, *Microsporum audouinii*, and *Trichophyton* sp. After autoclaving, potato dextrose agar (PDA) was incorporated into the EEHS at final concentrations of 5 mg/mL and 10 mg/mL, followed by plating onto Petri dishes. Tartaric acid at 1% and chloramphenicol at 0.1 g/L were added to the PDA. Subsequently, a 5-mm culture disc of each fungal strain tested was transferred to the center of the dishes. The discs were obtained from the edges of colonies previously grown for seven days on PDA. The plates were incubated at 25 ± 2°C for 72 h. After this period, the diameters of the fungal growth zones were measured [34]. Assays were performed in duplicate for each microorganism.

### Cytotoxic activity

#### Cell lines and culture conditions

The acute myeloid leukemia cell line Kasumi-1 was cultured in RPMI 1640 medium (Gibco; Rockville, MD, USA) supplemented with 10% fetal bovine serum (FBS) (CULTILAB, Brazil), 100 U/mL of penicillin, and 100 μg/mL of streptomycin, and it was incubated in a humidified atmosphere at 37°C and 5% CO_2_.

#### Assay with Annexin V and propidium iodide

The cytotoxic activity was evaluated using the method described by Paredes-Gamero et al. [35], with some modifications. Cells were plated onto 96-well microplates (10^5^ cells/mL) in RPMI 1640 supplemented with 10% FBS in the absence or presence of the EEHS (25–200 μg/mL) for 24 h in a humidified atmosphere at 37°C and 5% CO_2_. After this period, the cells were washed with PBS and resuspended in Annexin buffer (0.01 M HEPES, pH = 7.4, 0.14 M NaCl and 2.5 mM CaCl_2_). Annexin V conjugated with fluorescein isothiocyanate (FITC) and propidium iodide (PI) was added to each cell suspension according to the manufacturer's instructions, and each suspension was incubated for 20 min at room temperature. The stained cells were analyzed using an Accuri C6 flow cytometer (Becton Dickinson, NJ, USA) and Accuri C6 software (Becton Dickinson). A total of 4000 events were collected per sample.

#### Evaluation of the mitochondrial membrane potential

Changes in the mitochondrial membrane potential were assessed using 5,5’,6,6’-tetrachloro-1,1',3,3'-tetraethylbenzimidazolylcarbocyanine iodide (JC-1, Molecular Probes; Eugene, OR, USA) following the method described by de Moraes et al. [36]. JC-1 is a cationic marker with a membrane potential-dependent accumulation in the mitochondria, indicated by the change of the fluorescence emission from red (590 nm) to green (520 nm). The cells labeled red indicate a higher mitochondrial membrane potential, whereas those labeled green indicate a lower potential. To this end, Kasumi-1 cells were seeded in 24-well plates (10^5^ cells/mL) in medium containing 10% FBS and were treated with the IC_50_ (μg/mL) of the EEHS or carboxycyanide-4-(trifluoromethoxy)-phenylhydrazone (10 μM) (control) for 24 h in a humidified atmosphere at 37°C and 5% CO_2_. Subsequently, the cells were centrifuged and incubated with JC-1 (1 μg/mL) for 15 min at room temperature. Fluorescence was detected in an Accuri C6 flow cytometer (Becton Dickinson, USA) using Accuri C6 software (Becton Dickinson, USA). A total of 4000 events were collected per sample.

#### Effect of inhibitors on EEHS-induced cell death

Kasumi-1 cells were plated onto 96-well microplates (10^5^ cells/mL) containing RPMI 1640 supplemented with 10% FBS in the presence of 20 μM of the pan-caspase inhibitor Z-Val-Ala-Asp-(O-methyl)-fluoromethyl ketone (Z-VAD-FMK), 20 μM of necrosis inhibitor necrostatin-1 (NEC-1), 20 μM of cathepsin inhibitor trans-epoxysuccinyl-L-leucylamido-(4-guanidino) butane (E64), and 50 μM of reactive oxygen species inhibitor N-acetyl-L-cysteine (NAC), and it was incubated in a humidified atmosphere at 37°C and 5% CO_2_ for 60 min. Afterwards, 160 μg/mL of the EEHS was added to each sample, and the mixture was incubated for 24 h. Then, the cells were washed with PBS, resuspended in Annexin buffer (0.01 M HEPES, pH = 7.4, 0.14 M NaCl and 2.5 mM CaCl_2_) and incubated for 20 min at room temperature after the addition of annexin V-FITC and propidium iodide (PI) (Becton Dickinson, Franklin Lakes, NJ) according to the manufacturer's instructions. The analyses were performed using an Accuri C6 flow cytometer (Becton Dickinson) and Accuri C6 software (Becton Dickinson), with 4000 events collected per sample.

### Statistics

All data are show as the mean ± standard error of mean (SEM) and for statistical significant differences between the groups, using the *t-test* for comparison between two groups, using the Prism 6 GraphPad software. The results were considered significant when p * P < 0.05, ** P < 0.01 e *** P < 0.001.

## Results

### Chemical composition

**Fig 1:**
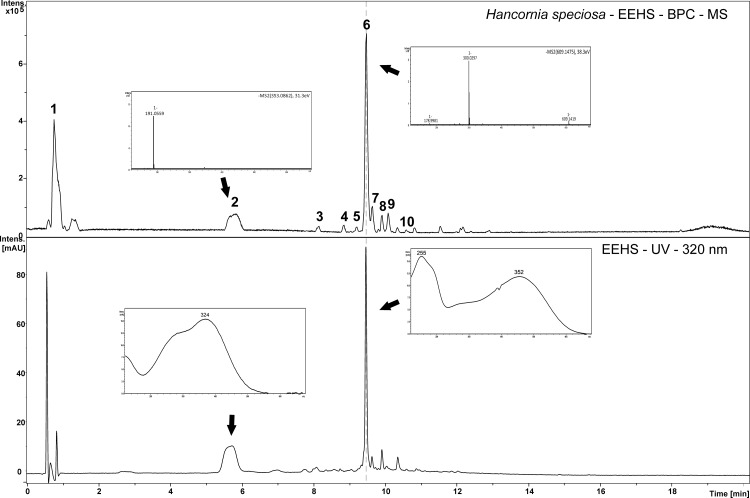
Chemical composition. Chromatograms obtained by HPLC-DAD-MS/MS (negative ion mode) indicating the presence of phenolic compounds in the EEHS. The enlarged figures correspond to the MS/MS spectra of the following main compounds: chlorogenic acid (peak 2) and rutin (peak 6).

The concentrations of phenolic compounds and total flavonoids in the EEHS were 179 ± 2.9 mg GAE/g of extract and 29 ± 1.1 mg QE/g of extract, respectively. The chemical profile of the extract was determined using HPLC-DAD-MS/MS (Fig 1 and Table 1) and indicated the presence of quinic acid (1), chlorogenic acid (2), catechin (3), rutin (6), and isoquercitrin (7), which were identified by comparison with authentic standards. In addition, two other heterosidic flavonoid derivatives were characterized, kaempferol-rutinoside (8), and their fragmentation patterns were similar to those of rutin but differed in the absence of a hydroxyl group in the flavonoid. They were identified through the fragment with an *m/z* of 285.0393 (C_15_H_9_O_6_). Compound 10 had a UV spectrum similar to that of catechin and was found at an *m/z* of 435.1282, consistent with the molecular formula C_21_H_23_O_10_ and corresponding to the addition of a pentose to catechin. These fragmentation patterns allowed the characterization of this molecule as a catechin-pentoside derivative.

**Table 1:** Phenolic compounds identified from the HPLC-DAD-MS/MS of the ethanolic extract of *Hancornia speciosa* leaves.

ID	RT [min]	Molecular Formula	[M-H]^-^	Error PPM	MS/MS	Compound
1	0.8	C_7_H_12_O_6_	191.0565	–2.1	—	Quinic acid*
2	5.8	C_16_H_18_O_9_	353.0878	1.5	191.0556; 173.044; 161.0228	Chlorogenic acid*
3	8.1	C_15_H_14_O_6_	289.0704	–2.8	—	Catechin*
4	8.8	C_16_H_20_O_10_	371.0967	–-3.0	249.0615	Unknown
5	9.2	C_17_H_22_O_10_	385.1132	–0.7	249.0578	Unknown
6	9.5	C_27_H_30_O_16_	609.1476	3.3	300.0303; 271.0251	Rutin*
7	9.6	C_21_H_20_O_12_	463.0867	–2.1	300.0265	Isoquercetrin*
8	9.9	C_27_H_30_O_15_	593.1494	–2.1	285.0393; 284.0313; 255.0291	Kaempferol-rutinoside
9	10.1	C_24_H_20_O_9_	451.1017	–2.7	341.0638; 231.0281; 217.0127; 189.0202; 177.0175	Unknown
10	10.6	C_21_H_24_O_10_	435.1282	–3.4	273.0779; 167.0310	Catechin-pentoside

* identified by comparison with an authentic standard

### Antioxidant activity

#### Scavenging of DPPH free radicals

The EEHS had a higher DPPH free radical-scavenging activity than did the lipophilic antioxidant standard BHT; however, the scavenging activity of the EEHS was lower than that of the hydrophilic antioxidant standard ascorbic acid. The IC_50_ of the EEHS was 7.1-fold lower than that of BHT and 3.2-fold higher than that of ascorbic acid. The maximum scavenging activity of the EEHS (in μg/mL) was 5-fold higher than that of ascorbic acid and 5-fold lower than that of BHT (Table 2).

**Table 2:** IC_50_ of the ethanolic extract of *Hancornia speciosa* Gomes leaves (EEHS) compared with the antioxidant standards ascorbic acid and butylated hydroxytoluene (BHT) along with the percentage of maximum scavenging activity of the free radical 2,2-diphenyl-1-picrylhydrazyl (DPPH).

DPPH	IC_50_ (μg/mL)	Maximum Activity	
		%	μg/mL
Ascorbic acid	2.9 ± 0.8	96.6 ± 0.3	10
BHT	66.1 ± 23.6	95.1 ± 0.5	500
EEHS	9.4 ± 0.8	94.8 ± 0.8	50

#### Protective effect of the EEHS against AAPH-induced hemolysis

**Fig 2:**
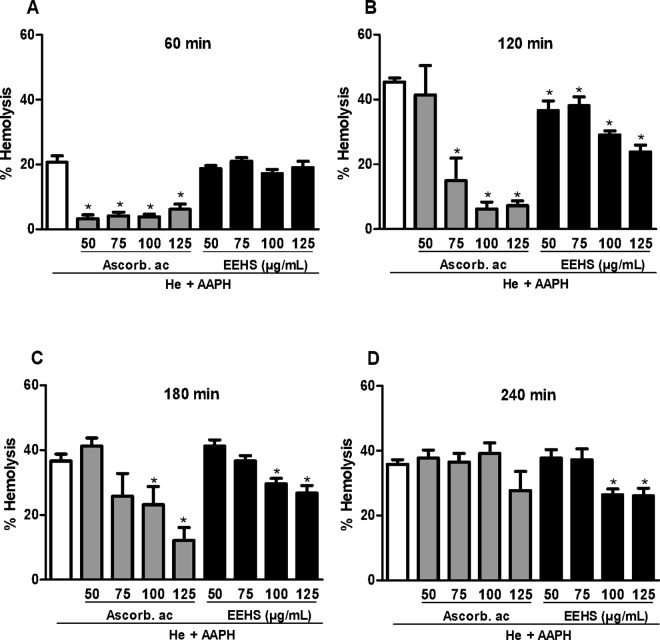
Effects of EEHS on the inhibition of hemolysis of human erythrocytes induced by AAPH. The groups were evaluated at (A) 60, (B) 120, (C) 180, and (D) 240 min of incubation. The controls consisted of an erythrocyte suspension (2.5%) incubated with AAPH alone. Values are expressed as the mean ± SEM of three independent experiments performed in duplicate. * P < 0.05 when the treated groups were compared with the AAPH group (erythrocytes incubated with AAPH alone) during the respective incubation periods.

The protective effect against AAPH-induced hemolysis was evaluated for the EEHS. With regard to the protection against AAPH-induced hemolysis, ascorbic acid prevented erythrocyte hemolysis within 180 minutes in a concentration-dependent manner (Fig 2A–2C). The EEHS protected erythrocytes incubated with AAPH for up to 240 min and decreased hemolysis by 20.1% and 21.4% at concentrations of 100 μg/mL and 125 μg/mL, respectively (Fig 2A–2D).

#### Inhibition of malondialdehyde production

**Fig 3:**
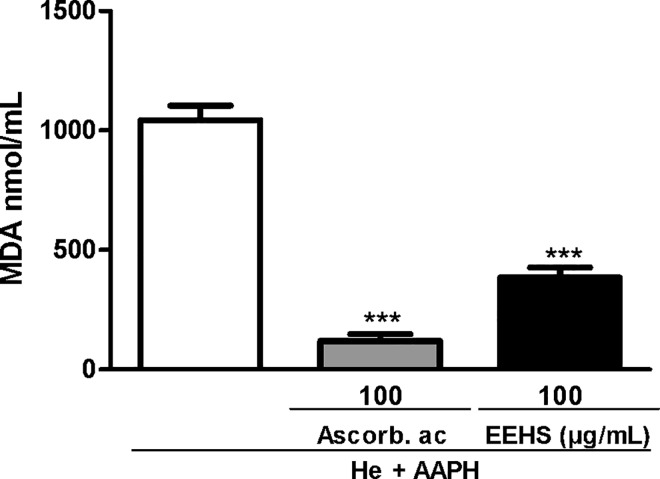
Effects of the EEHS on the inhibition of lipid peroxidation induced by AAPH. The control corresponds to erythrocytes incubated with AAPH alone. Values are expressed as the mean ± SEM of two independent experiments performed in duplicate. *** P < 0.001 when the treated groups were compared with the AAPH group (erythrocytes incubated with AAPH alone).

The antioxidant properties of the EEHS were also evaluated by determination of the inhibition of the production of MDA, which is the by-product of lipid peroxidation of erythrocyte membranes induced by AAPH. Treatment of erythrocytes with ascorbic acid and the EEHS at the lowest concentration able to inhibit hemolysis (100 μg/mL) for 3 h decreased the MDA levels by 88% and 63%, respectively, compared with the control group incubated with AAPH alone (Fig 3).

### Antimicrobial activity

#### Minimum inhibitory concentration

Table 3 shows that the EEHS was effective against all of the microorganisms evaluated, including a gram-positive strain (*S*. *aureus*) and a gram-negative strain (*P*. *mirabilis*). However, it presented bacteriostatic and fungistatic activity against a gram-negative strain (*K*. *pneumoniae*) and the yeast *Candida albicans*, respectively. In addition, all ATCC strains were more sensitive to the action of the EEHS compared with hospital strains, except for *K*. *pneumoniae* and C. *albicans*, which showed the same MIC.

**Table 3:** Minimal inhibitory concentration (MIC), minimum bactericidal concentration (MBC), and minimum fungicidal concentration (MFC) of the EEHS and controls.

	EEHS mg/mL		Gentamicin mg/mL		Amphotericin B mg/mL	
Microorganism	MIC	MBC/MFC	MIC	MBC	MIC	MFC
*S*. *aureus (ATCC 43300)*	0.78	3.12	0.0312	0.0625	-	-
*P*. *mirabilis (ATCC 25933)*	3.12	6.25	0.0625	0.0625	-	-
*K*. *pneumoniae (ATCC 13883)*	12.5	ND	0.00012	0.00012	-	-
*C*. *albicans (ATCC 10231)*	6.25	ND	-	-	0.0020	˃0.003
*S*. *aureus (ESA)*	3.12	6.25	0.125	0.125	-	-
*P*. *mirabilis (ESA)*	6.25	12.5	0.00049	0.00049	-	-
*K*. *pneumoniae (ESA)*	12.5	ND	0.0039	0.0039	-	-
*C*. *albicans (ESA)*	6.25	ND	-	-	0.0021	˃0.003

ATCC, American Type Culture Collection; ESA, Agricultural School, Bragança, Portugal.

ND, Not detected; -, Unvalued.

#### Fungal growth inhibition assay

**Fig 4:**
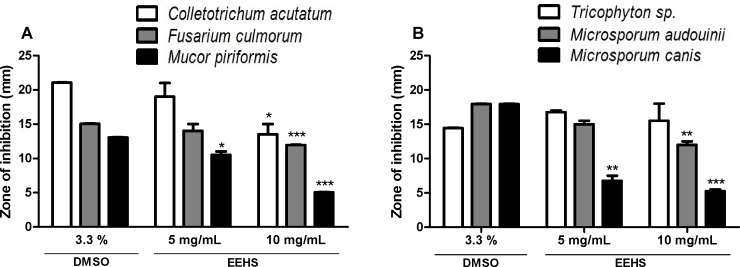
Zone of inhibition of filamentous fungi. Growth inhibition by the EEHS (5 mg/mL and 10 mg/mL) in the (A) filamentous fungal species *Colletotrichum acutatum* ESA12, *Fusarium culmorum* ESA23, and *Mucor piriformis* ESA43 and (B) dermatophytes *Microsporum canis* ESA28, *Microsporum audouinii*, and *Trichophyton* sp. (in mm). Values are expressed as the mean ± SEM of duplicate experiments. * P < 0.05, ** P < 0.01, and *** P < 0.001 when the treated groups were compared with the control group (3.3% DMSO).

The EEHS inhibited the growth of filamentous fungi and dermatophytes compared with the solvent (control). The filamentous fungal species most sensitive to the action of the EEHS was *M*. *piriformis*, which was inhibited at EEHS concentrations of 5 mg/mL and 10 mg/mL. The EEHS inhibited strains *F*. *culmorum* and *C*. *acutatum* only at the highest concentration evaluated (Fig 4A). The dermatophyte species most sensitive to the action of the EEHS was *M*. *canis*, which was inhibited at the concentrations of 5 mg/mL and 10 mg/mL. *M*. *audouinii* was sensitive to the 10 mg/mL concentration of the EEHS. At the concentrations tested, the EEHS did not affect the growth of *Trichophyton* sp. (Fig 4B).

### Cytotoxic activity

#### Cell death profile

**Fig 5:**
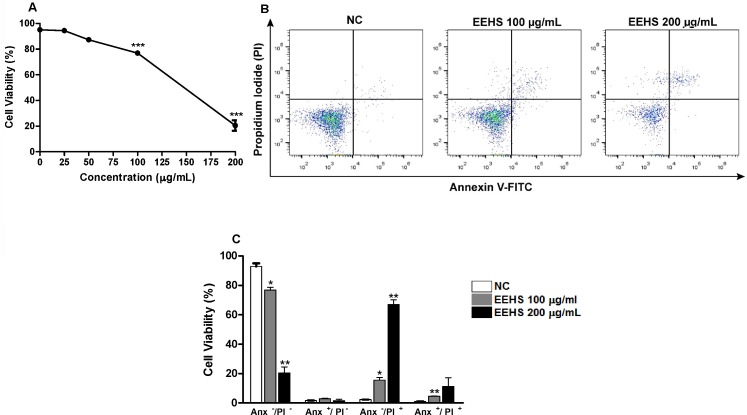
Cytotoxic activity of the EEHS in the acute myeloid leukemia cell line Kasumi-1. (A) Concentration-response curve (25–200 μg/mL). (B) Dot plots indicating the flow cytometry data of cells stained with propidium iodide (PI) and annexin-V-FITC (An) treated with a negative control (NC), 100 μg/mL of the EEHS or 200 μg/mL of the EEHS. The lower left quadrant shows viable cells (An^–^/PI^–^); the lower right quadrant shows apoptotic cells (An^+^/PI^–^); the upper left quadrant shows cells undergoing necrosis (An^–^/PI^+^); and the upper right quadrant shows cells in late apoptosis (An^+^/PI^+^). (C) Percentage of cell death obtained from dot plots of cells treated with NC, 100 μg/mL of the EEHS, or 200 μg/mL of the EEHS. * P < 0.05, ** P < 0.01 and *** P < 0.001 when the treated groups were compared with the control group.

The evaluation of the cytotoxicity of the EEHS in Kasumi-1 cells labeled with annexin V-FITC/PI indicated a decrease in the viability of tumor cells in late apoptosis (Fig 5B and 5C) by 21.6% and 78.7% at concentrations of 100 μg/mL and 200 μg/mL, respectively, and the IC_50_ was 160 μg/mL (Fig 5A).

#### Evaluation of the mitochondrial membrane potential

**Fig 6:**
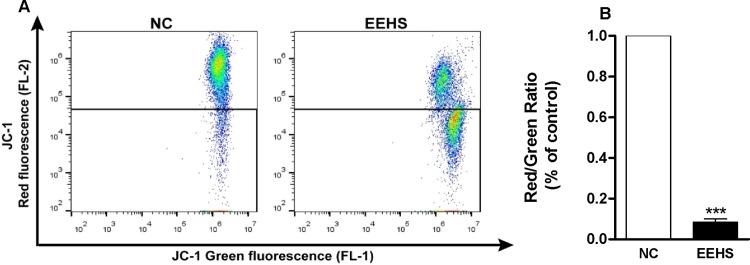
Estimation of the effect of the EEHS (160 μg/mL) on the mitochondrial membrane potential of the acute myeloid leukemia cell line Kasumi-1 by flow cytometry. (A) Dot plots of flow cytometric data indicating the pattern of cell staining using JC-1 in green (FL-1) and red (FL-2). (B) Percentage of change of the mitochondrial membrane potential obtained from dot plots. *** P < 0.001 when the treated groups were compared with the control group.

The analysis of the effects of the EEHS (IC_50_ = 160 μg/mL) on the mitochondrial membrane potential in Kasumi-1 cells in 24 h using the JC-1 fluorescent marker indicated a decrease in the potential by 98.4% (Fig 6A and 6B).

#### Effect of inhibitors on EEHS-induced cell death

**Fig 7:**
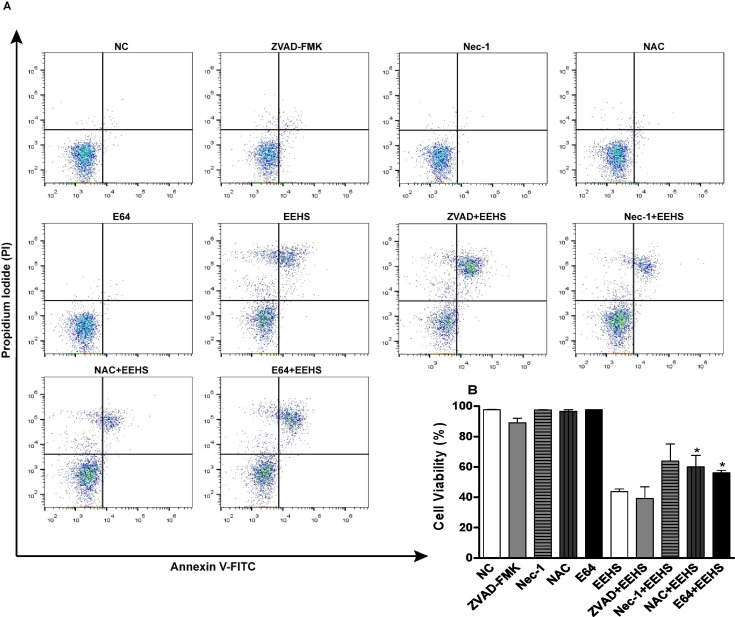
Cytotoxic activity of the EEHS in the acute myeloid leukemia cell line Kasumi-1 in the presence of cell death inhibitors. (A) Dot plots of the flow cytometry data of cells stained with propidium iodide and annexin-V-FITC in the presence of inhibitors Z-VAD-FMK, Nec-1, NAC, or E64 and in the presence of the EEHS combined with each of these inhibitors. (B) Percentage of cell viability obtained from dot plots at the EEHS concentration of 160 μg/mL. * P < 0.05 when the treated groups were compared with the control group.

The inhibitors E64 and NAC were effective in inhibiting the EEHS-induced death (IC_50_ = 160 μg/mL) of Kasumi-1 cells treated for 24 h. However, in this study, the apoptosis inhibitor Z-VAD-FMK and necrosis inhibitor NEC-1 were ineffective in inhibiting cell death (Fig 7A and 7B).

## Discussion

Plant extracts are naturally occurring products with complex chemical compositions. These compounds are responsible for the biological activity of the extracts and can act alone or synergistically. Previous phytochemical studies with leaves of *H*. *speciosa* identified several compounds, including L-(+)-bornesitol, quinic acid, chlorogenic acid, and flavonoids derived from kaempferol and rutin [4,37,38,39]. In this study, several phenolic derivatives were identified in the UV spectrum of the EEHS (Fig 1), and rutin along with chlorogenic acid were the major compounds in the extract. The amounts of total flavonoids and phenolic compounds in natural products are important parameters to be considered when assessing the quality and biological potential of natural products [40]. In this study, the concentration of total phenolic compounds in the EEHS was higher than that in the extract of *H*. *speciosa* fruit [41]. Phenolic compounds are hydrogen donors capable of directly scavenging free radicals and reducing oxidative damage [42,43], which makes them potent antioxidants. In other medicinal plant extracts, these compounds also activated endogenous antioxidant systems and inhibited the lipid peroxidation of human erythrocytes [2, 44]. Among the phenolic compounds, flavonoids are the most prominent because of their potent antioxidant activity [45].

The analysis of the direct scavenging of free radicals indicated that the EEHS was more effective than BHT, a synthetic antioxidant used in a wide variety of food products [46] and cosmetics [47]. The free radical-scavenging capacity of the EEHS was higher than that of the extracts of *H*. *speciosa* fruit [41], which may be attributed to the higher concentration of phenolic compounds in the leaf extract. In addition, the effects of the antioxidant activity in a biological model *in vitro* were evaluated in human erythrocytes subjected to lipid peroxidation by the action of free peroxyl radicals generated by the oxidizing agent AAPH. These radicals cause erythrocyte hemolysis via the oxidation of lipids and proteins of the cell membranes [48]. The EEHS demonstrated a sustained ability to protect against AAPH-induced hemolysis. The inhibition of lipid peroxidation was also determined by the quantification of the levels of MDA, which is a marker of oxidative damage to the lipids found in the erythrocyte membranes [49]. Umarani et al. [50] reported that the flavonoid rutin, also present in the EEHS, promoted the inhibition of lipid peroxidation in the heart tissue of rats subjected to oxidative stress. Henneberg et al. [51] reported the ability of rutin to sequester reactive oxygen species in human erythrocytes subjected to oxidative damage. Protection against lipid peroxidation promoted by the EEHS may be associated with its direct role in the scavenging of peroxyl radicals or the modulation of endogenous antioxidant mechanisms, including the activation of the enzymes glutathione, catalase, and superoxide dismutase [2,48].

In addition to the antioxidant activity, phenolic compounds have antimicrobial activity via several mechanisms, including adsorption to and disruption of microbial membranes, ion deprivation, enzyme interaction, and interaction with membrane transporters [52,53,54].

Amin et al. [22] found that quercetin derivatives, such as those present in the EEHS, were active against methicillin-resistant *S*. *aureus* strains. Previous studies with extracts of *H*. *speciosa* bark reported antimicrobial activity against gram-positive bacteria (*S*. *aureus*) and gram-negative bacteria (*H*. *pylori*) [3]. In this study, the EEHS presented antimicrobial activity against ATCC and hospital strains. The bactericidal action of the extract was observed against gram-positive and gram-negative bacteria. However, gram-negative bacteria are more resistant than gram-positive bacteria [55]. The bacterial cell wall, particularly in gram-negative bacteria, is an effective barrier against candidate drug molecules. This barrier is strongly polar and contains efflux pumps that act as a resistance mechanism, ejecting the compounds that pass through the outer membrane [56]. Al-Fatimi et al. [57] and Sahreen et al. [58] reported that plant extracts effective against gram-negative bacteria contained polar compounds that could interact with the chemical composition of the bacterial cell wall structure, thus promoting its effects.

The EEHS also presented fungistatic activity against the yeast *C*. *albicans* and inhibited the growth of filamentous fungi as well as dermatophytes. The presence of condensed tannins in the EEHS, particularly catechins, may explain the observed antifungal activity because these compounds can inactivate adhesion proteins, transporters, and enzymes [52].

Fungi promote superficial infections, which are common in elementary school children [59], and invasive infections, which are considered one of the leading causes of morbidity and mortality in immunocompromised patients [60]. The opportunistic nature of these infections increases the risk of infections in patients with prolonged neutropenia, lymphopenia, bone marrow transplants, and diabetes as well as those treated with corticosteroids [61].

Plants with antimicrobial activity also present cytotoxic activity by promoting death in various tumor cell lines [62] via different mechanisms. The primary routes of cell death are apoptosis, autophagy, and necrosis, and deaths by apoptosis and autophagy are considered programmed cell death mechanisms, whereas necrosis is considered an unregulated cell death mechanism [63]. However, cell death by necrosis may be regulated by a process known as necroptosis [64]. Previous studies indicated that the extracts of leaves, branches, fruit, and fruit latex of *H*. *speciosa* presented low toxicities towards the human tumor cell lines HCT-8 (colon carcinoma), MDA-MB-435 (melanoma), and SF-295 (glioblastoma) [65]. Moreover, our results indicated that the EEHS was effective in acute myeloid leukemia cells, and the cytotoxic activity was concentration-dependent and mediated by apoptosis. Martin et al. [66] found that the presence of phosphatidylserine in cell death assays with annexin V-FITC is considered an early event of apoptosis. Cell labeling with propidium iodide, which binds to DNA, is only observed during membrane damage, which occurs in late apoptosis or early necroptosis [67]. The flavonoids that are catechin and quercetin derivatives have potent cytotoxic activities and cause apoptosis using mitochondrial pathways in human leukemic cell lines, including monocytic leukemia (U937), erythroleukemia (K562), and promyelocytic leukemia (HL-60) [25]. Therefore, the presence of flavonoids in the EEHS can be directly associated with the cytotoxicity of the extract.

The EEHS decreased the mitochondrial membrane potential. Similarly, previous studies on cell viability reported the apoptotic activity of plant extracts in leukemic cell lines via reduction of the mitochondrial membrane potential [2,31,68].

Mitochondria-mediated apoptosis occurs in response to various death stimuli, including activation of tumor suppressor proteins and oncogenes, DNA damage, chemotherapeutic agents, nutritional deprivation, and ultraviolet radiation [69]. In these cases, apoptosis can occur via the intrinsic (mitochondrial-mediated) or extrinsic apoptotic pathways, both of which are caspase-dependent. Moreover, *in vitro* studies demonstrated that caspase-independent apoptosis can be regulated by lysosomes and endoplasmic reticulum [70]. Assessing the mechanism by which the EEHS promoted cell death, using cell death inhibitors, indicated the involvement of cysteine proteases (cathepsins). These results suggest that the EEHS promotes apoptosis via cathepsins because these enzymes induce apoptosis of tumor cells in a caspase-dependent and caspase-independent manner [71]. The EEHS promoted caspase-independent apoptosis considering that the pan-caspase inhibitor Z-VAD-FMK did not inhibit the cytotoxic activity of the extract. Cathepsins can also promote apoptosis by directly causing catalysis and proteolytic degradation of various substrates involved in cell death [72,73,74]. Antitumor agents such as vincristine, a naturally occurring alkaloid identified and isolated from plants of the genus *Vinca*, induce changes in the permeability of the lysosomal membrane and promote the release of cathepsins into the cytoplasm [75, 76]. Lysosomal cathepsins can act directly in the mitochondria by stimulating the release of cytochrome C and producing reactive oxygen species. The latter has direct and indirect effects on lysosomes [77] and causes progressive destabilization of the membranes of intracellular organelles, including lysosomes and mitochondria [78]. In this context, the EEHS strongly decreased the mitochondrial membrane potential and formation of reactive oxygen species, as shown by the inhibition of cell death using NAC, an inhibitor of these molecules.

Together, these results indicate that phenolic acids and flavonoids are present in the EEHS extract, and the antioxidant, antimicrobial, and cytotoxic activities of these compounds in acute myeloid leukemia cells are mediated by apoptosis via a decrease in the mitochondrial potential and cathepsin activation.
